# A phase II study of sequential carboplatin, paclitaxel and topotecan in patients with previously untreated advanced ovarian cancer

**DOI:** 10.1038/sj.bjc.6601618

**Published:** 2004-02-17

**Authors:** A E Guppy, A E Nelstrop, T Foster, R Agarwal, M J Seckl, G J S Rustin

**Affiliations:** 1 Department of Medical Oncology, The Clocktower, Mount Vernon Centre for Cancer Treatment, Rickmansworth Road, Northwood, Middlesex HA6 2RN, UK; 2 Charing Cross Hospital, London W6 8RF, UK

**Keywords:** ovarian cancer, sequential chemotherapy

## Abstract

We evaluated the sequential use of carboplatin, paclitaxel and topotecan in patients with advanced, previously untreated ovarian cancer. In total, 43 patients with advanced ovarian cancer and >1 cm residual disease were treated with sequential carboplatin (area-under-the-curve (AUC) 5 days 1 and 22), paclitaxel (175 mg m^−2^ days 43 and 64) and topotecan (1.5 mg m^−2^ daily for 5 days from days 85, 106, 127 and 148). Median age of patients was 61 years. Median follow-up was 22.2 months (range 0.76–50.6 months). In all, 34 (79%) patients received all eight cycles of treatment and nine (21%) withdrew. Of the 29 evaluable patients, 19 (66%) responded according to WHO and 30 of 36 (83%) patients according to CA125. The best overall response (CA125 and/or WHO) was 77% (33 of 43 patients). The response rates to sequential drugs based on >50% fall in CA125 were as follows: carboplatin, 77% (30 of 39 patients); paclitaxel, 65% (15 of 23 patients); topotecan, 38% (five of 13 patients). Two patients responded to paclitaxel and one to topotecan after failure to respond to preceding chemotherapy. Median survival and time to progression was 22.24 and 10.61 months, respectively. This study demonstrates that sequential chemotherapy with just two initial courses of carboplatin is a reasonable way to introduce new agents into first-line therapy for poor prognostic ovarian cancer patients.

Ovarian cancer remains the leading cause of death from gynaecological malignancy in Europe and North America (Pisani *et al*, 1999). Surgical cytoreduction followed by systemic chemotherapy forms the main stay of treatment for patients with advanced disease, with initial objective response rates ranging from 60 to 80% (Markman and Hoskins, 1992). Unfortunately, the majority of women with advanced disease relapse and the overall 5-year survival remain less than 30% (Averette and Donato, 1990). Consequently, alternative chemotherapy regimens are urgently required to improve these disappointing survival statistics.

Combination chemotherapy with paclitaxel and carboplatin or single-agent carboplatin are regarded as the standard of care as first-line treatment for ovarian cancer (NICE Guidelines, 2003). However, the improvement in survival of combination platinum-containing regimens over single-agent platinum has been questioned by two large randomised trials. The ICON3 trial (ICON Group, 2002) compared paclitaxel and carboplatin with a reference arm of either single-agent carboplatin or a combination of cyclophosphamide, adriamycin and cisplatin in 2074 patients, and showed no difference in overall survival between the reference and the paclitaxel-containing arms (HR=0.98, 95% CI 0.87–1.10). The GOG-132 trial (Muggia *et al*, 2000) compared single-agent paclitaxel and single-agent cisplatin with the two drugs combined in 648 patients and also showed no difference in overall survival between the two cisplatin-containing arms (HR=1.06, 95% CI 0.895–1.30).

There is also uncertainty in the literature as to the optimum number of treatment cycles (Lambert *et al*, 1997) and the optimum platinum dose (Ngan *et al*, 1989; Kaye *et al*, 1996). With specific regard to carboplatin dose intensity, two trials failed to show any significant survival benefit between patients treated with an area-under-the-curve (AUC) of 4 or 8 (Jakobsen *et al*, 1997) or AUC 6 or 12 (Gore *et al*, 1998).

Incorporating new, potentially noncross resistant agents into first-line combinations has the advantage of exposing tumour cells to all drugs simultaneously and theoretically preventing the emergence of drug-resistance clones. However, toxicity is often severe and doses need to be reduced. An alternative strategy involves sequential treatment with individual drugs. This approach has the advantage of eliminating the additive toxicity of combined drugs and also permits full dosages of each drug. Moreover, as the tumour becomes resistant to one drug, treatment with a nonresistant second drug should overcome the problem of resistant clones. Interestingly, *in vitro* fibroblast models with p53 mutations have been found to be hypersensitive to paclitaxel but resistant to platinum, while cell lines with normal p53 function are most sensitive to platinum (Wahl *et al*, 1996). Therefore, by using sequential treatment with platinum followed by a taxane, one would expect the population of p53-competent tumour cells to be eradicated by the initial platinum, leaving a second population of predominantly mutant p53 cells highly sensitive to the taxane.

Extending the platinum-free interval in recurrent ovarian cancer by using a noncross resistant chemotherapy agent at first relapse may have the advantage of increasing the response rate to platinum reinduction on further progression (Kavanagh *et al*, 1996). Within this study, therefore, we might expect an increased response rate to second-line platinum-containing regimens following sequential paclitaxel and topotecan.

There is some evidence from preclinical models that therapeutic synergy occurs when combining platinum with paclitaxel or topotecan (Kamazawa *et al*, 2000), and therefore a potential disadvantage of sequential therapy would be the loss of antitumour activity. This disadvantage, however, was not seen in a recent phase III trial comparing sequential single-agent doxorubicin and paclitaxel to combination doxorubicin and paclitaxel in metastatic breast cancer patients. Although inferior overall response rates and time to progression were seen in the sequential arm, there was no significant difference in overall survival (Sledge *et al*, 2003).

Topotecan, a topoisomerase-1 inhibitor, has been extensively tested in recurrent ovarian cancer and has shown a 13.4% response rate using a intravenous (i.v.) dose of 1.5 mg m^−2^ daily for 5 days (Gore *et al*, 2002). However, bone marrow suppression is considerable, limiting its use in combination with other chemotherapeutic agents.

This phase II trial was therefore designed to assess the feasibility and the response rates of sequential chemotherapy using the commonly used doses of carboplatin, paclitaxel and topotecan as first-line treatment for advanced ovarian cancer patients.

## PATIENTS AND METHODS

### Patient eligibility

All patients had histologically confirmed advanced epithelial ovarian cancer (FIGO stage IIB, IIIA, IIIB, IIIC or IV) and >1 cm residual disease at the completion of initial surgery. Diagnostic surgery had to be performed <6 weeks prior to entry to the study. Eligible patients had not received previous chemotherapy, radiotherapy or hormonal therapy, had a performance status of <2 (European Co-operative Oncology Group, ECOG Scale), were between 18 and 75 years of age, had a life expectancy of >12 weeks and were required to have adequate haematological (neutrophil count >1.5 × 10^9^ l^−1^; platelet count >100 × 10^9^ l^−1^; haemoglobin >9.0 g dl^−1^), renal (serum creatinine <2 × ULN, EDTA clearance >50 ml min^−1^) and hepatic (alkaline phosphatase and alanine transaminase <2.5 ULN if no liver metastases present or <5 × ULN if liver metastases are present) functions. Patients with uncontrolled infection, concurrent severe medical conditions, pre-existing motor or sensory neurotoxicity (>grade 2 according to National Cancer Institute-Common Toxicity Criteria (NCI-CTC)) and history of previous malignancies were excluded.

The protocol was approved by the Ethics Review Committees of the two cancer centres. Written informed consent was obtained from each patient before accrual.

### Study design

This was a phase II study conducted between two cancer centres. In all, 40 evaluable patients were required to show a 70% response rate using confidence intervals of 56–84%. The treatment consisted of sequential carboplatin given as an i.v. infusion over 60 min on days 1 and 22, followed by paclitaxel, 175 mg m^−2^ given over 3 h on days 43 and 64 and topotecan, 1.5 mg m^−2^ given over 30 min for 5 days from days 85, 106, 127 and 148. The dose of carboplatin was calculated according to the AUC method (Calvert *et al*, 1987), that is, 5(GFR+25) mg, where GFR is the glomerular filtration rate calculated using ^5^Cr EDTA. A full blood count was performed prior to each course of carboplatin and paclitaxel, but weekly during topotecan. Dose reductions were based on the day 22 blood count after carboplatin or paclitaxel, but on nadir counts after topotecan. Dose reductions of carboplatin and/or paclitaxel were carried out according to the following haematological toxicities: 10% dose reduction after recovery from grade 1 thrombocytopenia, or grade 2 neutropenia, 25% dose reduction after recovery from grade 2 thrombocytopenia, or grade 3 neutropenia and 50% after recovery from grade 3 thrombocytopenia and grade 4 neutropenia. The paclitaxel dose was also reduced by 50% if there was sustained grade 3 peripheral neuropathy. The daily infusion dose of topotecan was reduced by 0.25 mg m^−2^ day^−1^ if a neutrophil nadir of <0.5 × 10^9^ l^−1^ was associated with fever or infection or lasted >7 days or if a neutrophil nadir of 0.5–0.9 × 10^9^ l^−1^ lasted beyond day 28 of the treatment course or if there was a platelet nadir count of <25 × 10^9^ l^−1^. All patients continued on the same dose reduction for each drug for the remainder of the study. Study treatment was discontinued if haematological toxicity persisted after a maximum 2-week delay.

All patients had a physical examination, CA125 level, chest X-ray and abdominal/pelvic CT scan prior to the start of treatment. Disease was reassessed by repeat physical examinations and CA125 levels prior to each chemotherapy cycle and repeat CT scans on the completion of cycles 4 and 8 in those patients with measurable disease according to the World Health Organisation (WHO) criteria (WHO handbook, 1979).

### Response assessment

Tumour response was assessed in those patients with measurable disease using the World Health Organisation criteria. To increase the proportion of evaluable patients those with CA 125 levels >120 U ml^−1^ postsurgery and prechemotherapy, were also assessed according to precise CA125 criteria. A CA125 response had occurred if there was either a 50 or 75% decrease in CA125 levels using a ‘CA125 evaluation’ computer programme (Rustin *et al*, 1996a; Rustin *et al*, 2000). A 50% response occurred if after two initial elevated samples there had been a 50% decrease of serum CA 125 levels, confirmed by a fourth sample. A 75% response occurred if there has been a serial decrease of serum CA 125 levels over three samples of at least 75%. In both 50 and 75% definitions, the final sample had to be at least 28 days after the previous sample.

Progression was defined either by WHO criteria or by CA 125. The previously validated CA125 progression criteria (Rustin *et al*, 1996b, 2001; Vergote *et al*, 2000) is defined as either a doubling of the CA 125 levels from the nadir value achieved during previous therapy or a doubling from the upper limit of normal. (Value defined at local laboratories.) In each definition, the rise in CA125 was confirmed with a second sample. The date of progression was the date of the earliest event indicating progression either according to CA125 or WHO criteria. Progression-free survival was defined as the time between the start of treatment and the date of first documented progression. Overall survival was the time between the start of treatment and death.

## RESULTS

In total, 43 patients were entered into this phase II study across two cancer centres between April 1998 and June 2001. The patient characteristics of all patients are listed in Table 1

**Table 1:** Patient characteristics

	**Patient no.**	**%**
*Age (years)*		
Median	61	
Range	40–79	
		
*Performance status*		
0	12	28
1	22	56
2	9	16
		
*FIGO stage*		
II	1	2
III	28	65
IV	14	33
		
*Cell type*		
Serous	25	58
Endometrioid	6	14
Mucinous	2	5
Other	10	23
		
Measurable disease	29	67
		
*Type of surgery*		
Biopsy only	11	26
Debulk (>1 cm at close)	32	75

. A total of 34 (79%) patients completed the study. Of the nine (21%) patients who withdrew, four had progressive disease, three died on treatment (one due to a cerebrovascular event, one due to neutropenic sepsis, one due to peritonitis), one had a severe allergic reaction to paclitaxel and one refused further chemotherapy after interval debulking surgery.

### Toxicity

The worse grade of toxicity that was experienced by each patient throughout each course of chemotherapy was recorded (Table 2

**Table 2:** Grade 3+4 toxicity (number and % of patients and courses with toxicity)

	**Carboplatin**				**Paclitaxel**				**Topotecan**			
	**Total patients, *N*=43**	**%**	**Total courses, *N*=85**	**%**	**Total patients, *N*=41**	**%**	**Total courses, *N*=80**	**%**	**Total patients, *N*=38**	**%**	**Total courses, *N*=145**	**%**
*Nonhaematological*												
Nausea/vomiting	3	7	2	2	0	0	0	0	1	3	1	1
Mucositis	0	0	0	0	1	2	1	1	0	0	0	0
Diarrhoea	1	2	1	1	0	0	0	0	2	5	2	1
Alopecia	0	0	0	0	15	37	22	28	18	47	72	50
Thromboembolic event	2	5	2	2	1	2	2	3	0	0	0	0
												
*Haematological*												
Anaemia	1	2	1	1	2	5	2	3	6	16	6	4
Neutropenia	2	5	0	0	2	5	3	4	31	82	88	61
Neutropenic sepsis	2	5	2	2	2	5	2	3	5	13	8	6
Thrombocytopenia	1	2	1	1	0	0	1	1	4	11	5	3

). Grade 3 or 4 haematological toxicity was low during both carboplatin and paclitaxel cycles but high during topotecan. Nonhaematological toxicity was minimal. In all, 16 patients (37%) had at least one dose delay and 10 patients (23%) required at least one dose reduction.

### Response

The median duration of follow-up in this study was 22.2. months (range: 0.76–50.6 months). In all, 36 patients were evaluable for response by CA 125 criteria and 30 of these achieved a response, giving an overall response rate of 83.3%. Seven patients were nonevaluable for response according to CA125 criteria, five due to less than three samples being available for analysis and two in whom all CA 125 values were <40 U ml^−1^. A total of 29 patients had measurable disease according to standard criteria and 19 of these achieved a response, giving an overall response rate of 65.5%. Three patients were not evaluable by either CA 125 or standard criteria, leaving 40 patients assessable by combined CA 125 and standard criteria. Of these, 30 patients responded giving an overall response rate of 75% in evaluable patients and 70% among all patients treated. Response rates according to a 50% fall in CA125 levels for evaluable patients were analysed for each individual drug. Of 39 evaluable patients, 30 responded to carboplatin (76.9%), 15 of 23 evaluable patients responded to paclitaxel (65.2%) and five of 13 responded to topotecan (38.4%). Six patients progressed on treatment according to standard criteria (three progressed on the interval scan after receiving carboplatin and paclitaxel, and three had stable disease on the first scan, but showed progression by the end of treatment scan) giving an overall progression rate of 14%. Surgery also contributed to the CA125 response. Therefore, only patients with most resistant disease had persistently elevated CA125 levels on commencement of paclitaxel or topotecan.

**Figure 1:**
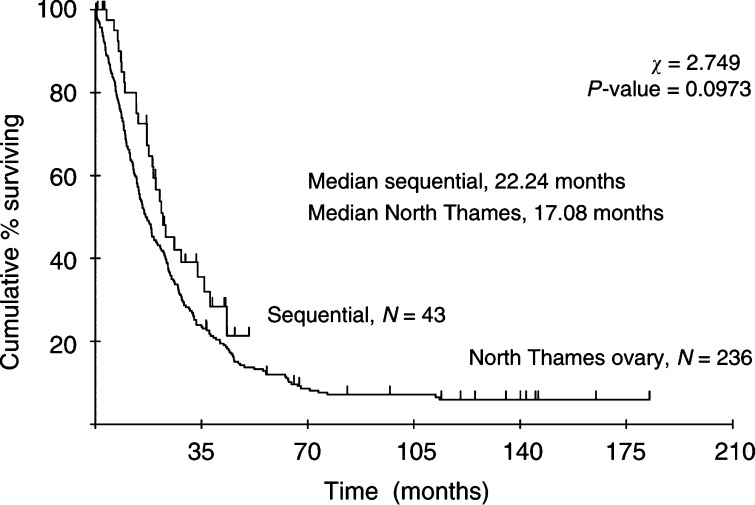
Overall survival in study patients compared to historical matched group from North Thames Ovary Trials 3+4 (>2 cm and inoperable).

The median overall survival was 22.2 months (Figure 1

). Survival in the study population is compared in these figures with that of a matched group of patients from two previous North Thames ovary trials and found to be similar (Lambert *et al*, 1993; Lambert *et al*, 1997). In this group, all patients had inoperable disease or measurable disease >2 cm at the end of surgery and received between five and eight cycles of carboplatin. The progression-free survival using both CA 125 and standard criteria to define progression was 10.61 months in the current trial compared to 10.45 months in the matched groups from the North Thames trials.

In the present study, 21 patients from the initial response group relapsed and went on to receive second-line chemotherapy (17 patients received single-agent carboplatin, two received carboplatin and paclitaxel, one received weekly cisplatin and etoposide (van der Burg *et al*, 2002) and one received altretamine). Seven of 14 evaluable patients responded to second-line platinum-containing chemotherapy according to CA125 criteria. Two of four evaluable patients relapsing within 6 months of completing carboplatin responded, while five of 10 evaluable patients relapsing beyond 6 months responded. The majority of patients (75%) received second-line chemotherapy 6 months or more from their initial carboplatin.

## DISCUSSION

To our knowledge this is the first published phase II trial of sequential chemotherapy in advanced ovarian cancer patients. However, in the previously reported Gynaecologic Oncology Trial (GOG-132), the vast majority of patients (85%) who relapsed on the single-agent arms went on to receive the other drug, thereby effectively receiving sequential carboplatin and paclitaxel chemotherapy (Muggia *et al*, 2000). Comparison of the patient characteristics from this trial with those of the above study show that although the two groups appear similar, the overall and progression-free survival are slightly inferior for the current phase II study. Reasons for this difference may be reflected in the extent of surgery undertaken or the amount of platinum chemotherapy each patient received. In both studies all patients were suboptimally debulked, but in the current trial, 25% of patients were only able to have a diagnostic biopsy at laparotomy.

Comparison of the survival data from our study with that of a matched population of patients with suboptimally debulked, advanced ovarian cancer, all of whom received between five and eight cycles of carboplatin (Lambert *et al*, 1993, 1997), were identical. Therefore, it is plausible that patients in the current study were not disadvantaged by receiving only two cycles of carboplatin. This trial was planned at a time when the GOG were running the 132 trial with single-agent paclitaxel as one arm, which was later shown to have equivalent survival to the platinum-containing arms (Muggia *et al*, 2000), implying that initial platinum therapy is not essential. Rather than give just six cycles of paclitaxel or six cycles of carboplatin as in ICON 3 (ICON group 2002), it seemed reasonable to give just two courses carboplatin prior to two courses of paclitaxel and then to follow this with topotecan. This is the true test of sequential therapy as if the standard total dose of carboplatin is given, adding other drugs later leads to greatly prolonged therapy, which could be almost considered maintenance therapy. Furthermore very few patients would have any evaluable disease left after completing full dose carboplatin. It is refreshing that at a time when most oncologists wish to give more intense combination therapy and are extending the number of courses, a less intense regimen dose appears not to be deleterious. Single-agent topotecan has been investigated extensively for its use in relapsed ovarian cancer, with response rates in platinum-sensitive patients ranging between 13 and 33% (Creemers *et al*, 1996; ten Bokkel Huinink *et al*, 1997; Bookman *et al*, 1998; Gore *et al*, 2002). Much work has also been carried out on whether using topotecan in first relapse can increase the platinum-free interval, and thus subsequent responses rates to platinum-containing regimens. One might speculate therefore that sequential use of topotecan may also increase the treatment-free interval as well as the response rate to second-line carboplatin. However, in this study although 15 out of 21 (71%) patients who initially responded relapsed 6 months or more after receiving carboplatin, the response rates to second-line platinum were 50% according to CA125 criteria in both platinum-resistant and platinum-responsive groups of patients. These responses are similar to those seen in previous studies so it is impossible to deduce that this response rate was affected by the low dose of initial carboplatin or the prolongation of platinum-free interval by the paclitaxel and topotecan.

Individual drug response rates were analysed according to a 50% fall in CA125 levels and found to deteriorate with each sequential drug. However, this is probably best explained by the decline in the number of evaluable patients required for the CA125 response criteria for each individual drug as the study progressed.

Toxicity of this regimen was acceptable. Although a high proportion of patients experienced haematological toxicity while on topotecan, (82% of patients had grade 3 or 4 neutropenia) only a small proportion (13%) required admission for neutropenic sepsis. In addition, dose reductions were only necessary in 10 (23%) patients.

Several randomised trials are currently investigating sequential chemotherapy as first-line treatment for ovarian cancer. The recently closed SCOTROC (Scottish Randomised Trials in Ovarian Cancer) 2A trial randomised 125 patients to receive four cycles of carboplatin, AUC 7, q21d followed by either four cycles docetaxel 100 mg m^−2^ q21d, four cycles docetaxel 75 mg m^−2^ (d8) plus gemcitabine 1250 mg m^−2^ (d1/8) q21d or 12 weeks docetaxel 25 mg m^−2^ plus gemcitabine 800 mg m^−2^ q1wk. Survival data are not yet mature; however, preliminary toxicity data suggested that carboplatin followed by subsequent three-weekly docetaxel with or without gemcitabine is feasible and safe. The weekly docetaxel and gemcitabine arm, however, produced significant delays and dose reductions (Rustin *et al*, 2002). The International Collaborative Ovarian Neoplasm and Gynaecologic Oncology Group trial groups are also currently investigating sequential chemotherapy. In this trial (GOG-0182, MRC-ICON5) patients will be randomised between the reference arm of eight cycles paclitaxel and carboplatin, and triplet (gemcitabine or pegylated liposomal doxorubicin with carboplatin and paclitaxel) or sequential doublet (gemcitabine or topotecan with carboplatin followed by carboplatin and paclitaxel) combinations. Approximately 6000 patients are required in this complex study in order to detect a significant difference between each experimental arm and the reference arm.

Both studies will use the previously validated CA125 criteria (Rustin *et al*, 1996a, 1996b) in addition to standard criteria to assess response and overall survival. The arguments for assessing response by either standard or CA125 criteria in ovarian cancer have been convincingly made (Rustin 2003). Our study demonstrates that analysing CA125 response to each individual drug is a logical and quick means of determining the response rate of each drug incorporated into the regimen.

In both of the above trials, patients receive a dose of carboplatin equivalent to that given in standard single-agent or combination regimens. The results of our trial suggest, however, that less carboplatin can be safely given, allowing larger doses of new agents to be tested. This phase II study has therefore shown that sequential carboplatin followed by paclitaxel and topotecan is a well-tolerated and effective regimen for advanced ovarian cancer. This approach is being further investigated in ongoing randomised clinical trials.
